# College and Hospital Notes

**Published:** 1909-06

**Authors:** 


### COLLEGE AND HOSPITAL NOTES.

Caroline Rest, a sanatorium for convalescing mothers who are poor, was formerly opened at Hartsdale, Westchester County, May 5, 1909. Four hundred invited guests, of whom most were women, attended the exercises. Talk on the train had been light and free, but there was something solemn about the dedication of Caroline Rest, established to meet a pathetic need, as a memorial to Caroline Schrader.

R. Fulton Cutting, president of the board of managers, presided. He told how George H. F. Schrader, son of Caroline Schrader, was touched by the fact that many poor mothers were compelled to return to the factory or to the care of the household within a week after childbirth. He therefore caused to be erected the building, beautiful in appointments and location, that was opened recently. It was deeded by Mr. Schrader to the New York Association for Improving the Condition of the Poor.

Robert W. Hebbard, Commissioner of Public Charities of New York, said the building was ideal for the purposes it was designed to meet, and that it was superior in detail to any building erected by the city for charitable purposes. Other speakers were Dr. John Winters Brannan, president of Bellevue and Allied Hospitals, and the Rev. Dr. Dennis J. McMahon.

The building can accommodate ninety persons, who, it is intended, shall be divided into three equal classes—mothers, children under eight years old and babies. The house, two and a half acres of ground and the endowment fund represent, it is said, a gift of $500,000 by Mr. Schrader, who, by the way, was too modest to be present. An officer of the association said Mr. Schrader had hurriedly departed for Europe to escape hearing his praises spoken.

Dr. McMahon said: "Caroline Rest is not only a sanatorium for mothers with infants, but a school where, under the most favorable conditions, mothers of the poor will receive instruction in the duties of motherhood, in personal hygiene, the care of their children and the right conduct of their homes."

The work at Hartsdale will be supplemented in this city by the Caroline Rest nurses, who, it is said, will visit mothers before and after confinement, and through instruction and special care promote their health and that of their children.

Members of the board of managers are Robert Shaw Minturn, Frederick Trevor Hill, John G. Agar, the Rev. Dr. John B. Devins, Leonard P. Opdycke, Percy R. Pyne, De Witt J. Seligman, R. Bayard Cutting and Isaac Townsend.

---

A NEUROLOGICAL institute for the treatment and study of nervous disorders is to be founded in New York, through the efforts of two local specialists in nervous diseases, and the subscriptions of a number of wealthy men. More than $100,000 has already been subscribed, and the construction of a building will begin as soon as a suitable site can be procured.

---

THE big four-story granite and graystone staff house for the internes of the City Hospital, on Blackwell's Island, New York, was formally opened April 15, 1909. The building is known as Janeway Hall, and will be the home of the physicians connected with the City Hospital. It is named in honor of Dr. Edward G. Janeway, who attended the exercises. The new building is 75 by 35 feet in size, and cost $75,000. It has twenty-six sleeping rooms, one for each doctor; a library, a staff room and a large dining room. Doctors of the city hospital now boast that they have the finest staff house of any hospital in the city.

---

A CONTAGION pavilion is to be built on the site of the Kimberly Cottage by the trustees of the Buffalo General Hospital. It will be one of the finest buildings of its size and kind in the country.

The trustees of the hospital have decided to bear the expense among themselves. The pavilion will fill a much needed place. It will be used primarily for patients who contract contagious diseases while patients at the General.

The Kimberly Cottage has outgrown its usefulness and will be torn down. Work on the new building will begin soon. The accommodations of the pavilion will be of the best. There are to be eight rooms for patients and others for nurses and attendants. Each will have toilet and showers, and may be arranged en suite.

The building will have four entrances and two exits. The exterior will be of buff brick, like the other buildings. In the basement will be a huge sterilizer.

---

A COURSE S 43-44, will be given in the Summer School, Columbia University, Department of Chemistry, New York, during the six weeks, July 7 to August 18, 1909. This course can be so modified as to include the study of those important organic substances which are of interest to medical men. The subject will be treated from the organic chemistry side and will include the results of the latest investigations. The following classes of compounds will be considered: sugars and carbohydrates, fats, amino acids and the hydrolysis of proteids, the uric acid group, alkaloids, essential oils, the artificial remedies and the bearing of their chemical constitution on their physiological function, etc. A series of eighteen lectures will accompany the course, if the attendance is sufficient to warrant it.

The following other courses will also be given: S E—Elementary physiological chemistry. S F—Chemistry of nutrition. S 171a—Organic and sanitary analysis. S 37—Elementary physical chemistry.

Further information concerning any of these courses can be obtained from the Summer School Announcement, and by writing to Professor J. Livingston R. Morgan.

